# Our surgical experience in foramen magnum meningiomas: clinical series of 11 cases

**DOI:** 10.11604/pamj.2019.34.5.17536

**Published:** 2019-09-03

**Authors:** Emre Bilgin, Gökhan Çavus, Vedat Açik, Ali Arslan, Semih Kivanç Olguner, Ismail Istemen, Yurdal Gezercan, Ali Ihsan Ökten

**Affiliations:** 1 Adana City Training and Research Hospital, Neurosurgery Clinic, Adana, Turkey

**Keywords:** Foramen magnum, meningioma, suboccipital, craniotomy, C1 laminoplastY

## Abstract

**Introduction:**

We aimed to discuss surgical approaches and results that we applied foramen magnum meningiomas.

**Methods:**

We retrospectively investigated 11 foramen magnum meningioma cases, who had been operated between the dates of February 2012 and March 2017.

**Results:**

Eight of the patients were females and 3 of the patients were males, the age range was 32-75 and the age average was 60.8. 5 of the tumors were anatomically localized as posterolateral, 2 of them were localized as anterolateral, 2 of them were localized as lateral and 2 of them were localized as anterior according to the brain stem or spinal cord. Posterior far lateral (4 patients) approach including C1 laminoplasty (7 patients) and 1/3 condyle resection was surgically applied to the patients with median suboccipital craniotomy. Gross total excision was applied to 82% of the patients (9 patients) and subtotal mass excision was applied to 18% (2 patients) of the patients. The most frequent post-operative complications were temporary lower cranial nerve (CN IX and X ) palsy in our 2 anterior localized cases (18%) and also cerebrospinal fluid (CSF) fistula in our 1 anterior localized case with difficulty in swallowing (dysphagia). Karnofsky scores of the patients, who were followed for 18 months in post-operative 12 and 48 months of average, in the last follow-up were 80 and no post-operative mortality occurred.

**Conclusion:**

Posterior midline suboccipital and far lateral approaches that we apply in our own series were appropriate approaches for foramen magnum meningiomas.

## Introduction

**Figure 1:**
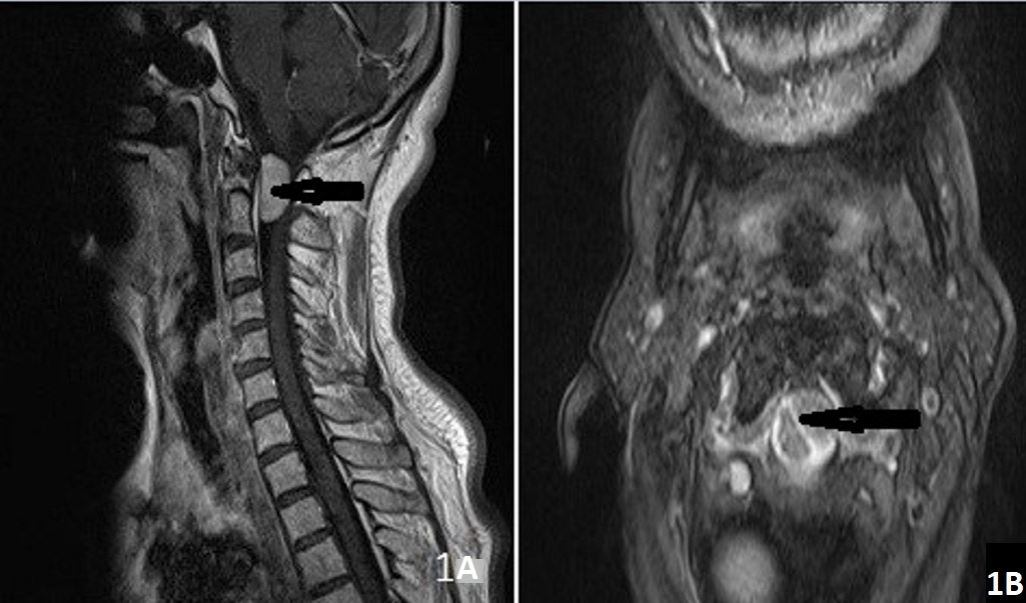
62 years old female patient (A) pre-operative sagittal MRI, (B) pre-operative axial MRI. Black arrow indicates tumor

Meningiomas are generally benign, and they are the tumors with good prognosis. Foramen magnum meningiomas constitute 1.8-3.2% of all meningiomas [1-4]. They are observed more frequently in fifth and sixth decades [5-8]. Their surgical methods are quite difficult and complex as they grow by pushing forward the important vascular structures such as lower cranial nerves and vertebrobasilar complex [3,5,6,8-10]. Joint stability between occipital bone, 1^st^ cervical spine (C1) and 2^nd^ cervical spine (C2) may be lost since they may cause craniocervical junction bone anatomy changes as they grow silently [7,10]. They are frequently reaching to large sizes when they are diagnosed (Figure 1). We primarily used posterior suboccipital craniotomy and C1 laminoplasty as a surgical approach and secondly far lateral approach including condyle resection for tumor resection in our study. We attempted to discuss the surgical approaches we apply and the results of these approaches on the rates of tumor removal by presenting our experience related to foramen magnum tumors in the last 7 years.

## Methods

**Figure 2:**
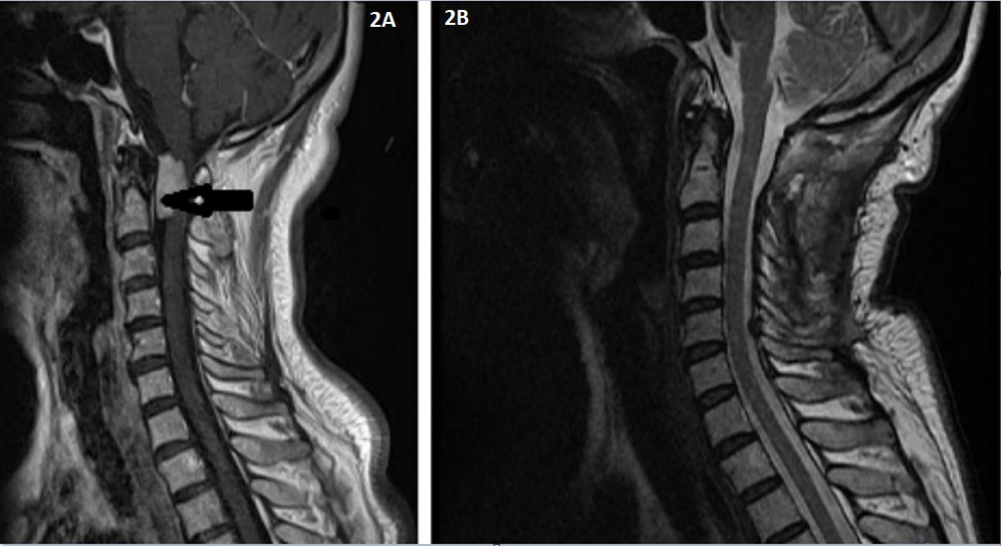
68 years old female patient (A) pre-operative sagittal MRI, (B) post-operative sagittal MRI. Black arrow indicates tumor

Surgical treatments were applied to 11 foramen magnum meningioma cases between the dates of February 2012 and March 2017 in our clinic. Patient files, clinical and surgical notes and records, radiological investigations, pathology results were retrospectively inspected. Surgical approaches applied and results and complications of these surgical approaches were evaluated by comparing to the literature by taking into consideration the demographic information, ages, genders, complaints, symptoms, clinical findings, neurologic examination, radiological findings, tumor morphology and localizations of 11 patients detected. Pre-operative magnetic resonance imaging (MRI) and computerized tomography (CT) were utilized. The tumors were classified as anterior, anterolateral, lateral, posterolateral and posterior according to medulla in axial plan in pre-operative MRI. (Figure 2). Pre-operative karnofsky performance scales (0-death 100-very good health) were used in order to evaluate the life quality of the patients and visual analog scales (0-no pain, 10-very severe pain) (VAS) were used in order to rate the neck pains. Complete removal of tumor with its capsula was evaluated as gross total tumor excision in our study. Karnofsky and VAS were repeated for our post-surgery results. 72.7% (8 patients) of 11 cases were females and 27.3% (3 patients) were males. Age ranges were 32-75 and age average was 60.8. The start of the symptoms was 14 months in average between 6 and 36 months. The start complaint and symptom were headache and neck pain, and they were observed 100% (11 patients) in all patients. While average of pre-operative average visual analog scale (VAS) values for neck pain was 6, Karnofsky performance scale average value was 70. While 18% (2 patients) had ataxia, 18% (2 patients) had weakness in arms, 18% (2 patients) had quadriparesis, 9% (1 patient) had difficulty of swallowing, neurological examination in 37% (4 patients) was normal. Demographic information, clinical, radiologic features, surgical approach applied and complications, pre-operative and post-operative karnofsky and VAS of 11 patients are summarized in Table 1.

**Table 1:** Demographic information, clinical, radiologic features, surgical approach applied and complications, pre-operative and post-operative karnofsky and VAS of 11 patients

Patient	Age/Gender	Tumor Localization	Tumor Resection	Surgical Approach	Complication	Karnofsky preop/postop	VAS Preop/postop
1	51y,M	Lateral	Gross total	Posterior midline	--	80/90	6/3
2	45y,M	Anterior	Subtotal	Far Lateral	New lower cranial paralysis (IX,X) dysphagia	60/80	4/6
3	35y,M	Posterolateral	Gross total	Posterior midline	--	90/90	5/4
4	72y,F	Posterolateral	Gross total	Posterior midline	--	70/80	6/4
5	68y,F	Posterolateral	Gross total	Posterior midline		70/90	5/4
6	54y,F	Posterolateral	Gross total	Posterior midline	--	80/80	7/4
7	32y,F	Anterolateral	Gross total	Far lateral	--	70/90	5/7
8	75y,F	Anterolateral	Gross total	Far lateral	New lower cranial paralysis (IX,X) dysphagia	60/80	7/5
9	51y,F	Anterior	Subtotal	Far lateral	CSF fistula	70/80	5/6
10	45y,F	Lateral	Gross total	Posterior midline	--	80/90	5/5
11	55y,F	Posterolateral	Gross total	Posterior midline	--	70/80	6/5

**Figure 3:**
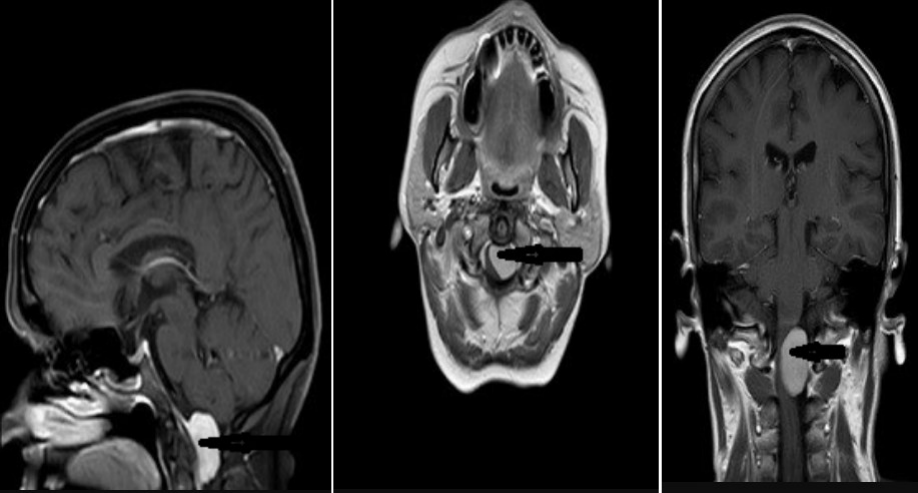
54 years old female patient pre-op MRI images and back arrow indicates tumor

**Figure 4:**
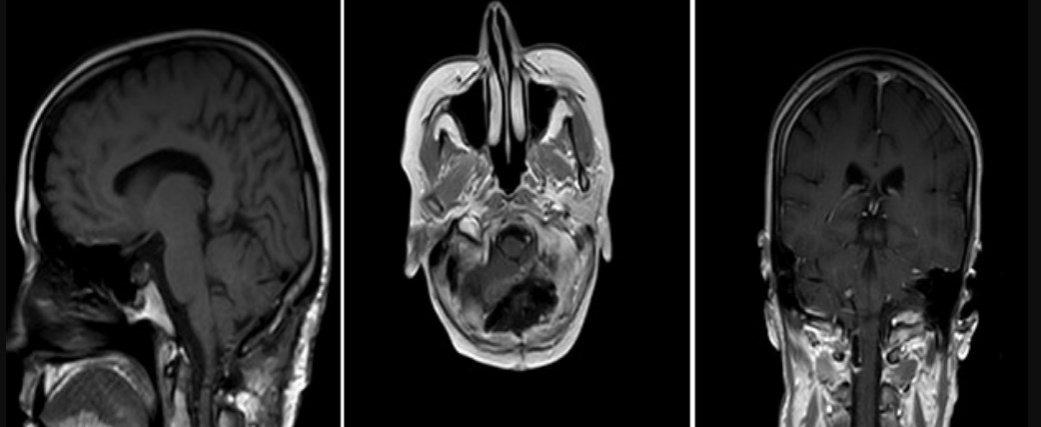
54 years old female patient post-op MRI images

5 (46%) of them were localized posterolateral, 2 (18%) of them were localized anterolateral, 2 (18%) of them were localized lateral, 2 (18%) of them were localized anterior (Table 2). While posterior median suboccipital craniotomy and C1 laminoplasty were made to 7 patients (64%) in total with posterolateral and lateral localization, posterior far lateral approach including 1/3 condyle resection was applied to 4 patients (36%) in total with anterolateral and anterior localization. While gross total mass excision was made to 9 patients (82%), subtotal mass excision could be made to 2 patients (18%) with anterior localization due to cohesion of tumor to cranial nerves (IX,X) and vascular structures (Table 2). While temporary lower cranial nerve involvement and difficulty in swallowing are observed in 1 patient (18%) for each anterior and anterolateral localization that we applied far lateral approach in post-operative period, CSF leakage developed in 1 patient (9%) with anterior localization that we applied far lateral approach. No mortality occurred. Post-operative karnofsky performance scale average was 80. Pathologies of all patients were WHO grade I meningiomas and 80% were meningothelial type and 20% were psammomatous type. 18 months of follow-up was performed in average in post-operative 12 and 48 months of period. No recurrence occurred. Neck pain VAS values in 2 patients with anterior localizations, to whom we only performed condyle excision by far lateral approach and we did't perform C1 laminoplasty, didn't regress in post-operative 6th month follow-ups. Each post-operative patient was performed MRI and evaluated in terms of residue, bleeding and edema. MRI was repeated in 1^st^month, 6^th^ month and 1^st^ year for residual tumor growth and pseudomeningocoele follow-up. Stable patients were received to MRI follow-up in 1 year of intervals (Figure 3, Figure 4).

**Table 2:** Genders, complaints, neurologic examination, imaging, surgical approach, tumor excision, tumor localization number and percentages

FORAMEN MAGNUM MENINGIOMA		NUMBER	PERCENTAGE %
**GENDER**	Male	3	27.3 %
	Female	8	72.7 %
**COMPLAINT**	Headache	11	100%
	Neck pain	11	100%
	Difficulty in swallowing	1	9%
	Imbalance in walking	2	18%
	Weakness in arms	2	18%
	Weakness in arms and legs	2	18%
**NEUROLOGICAL EXAMINATION**	Normal	4	37%
	Lower cranial nerve paralysis	1	9%
	Upper extremity weakness	2	18%
	Ataxia	2	18%
	Quadriparesis	2	18%
**IMAGING METHODS**	Mri	11	100%
**SURGICAL APPROACH**	Posterior midline suboccipital craniotomy and c1 laminoplasty	7	64%
	Far lateral	4	36%
**TUMOR EXCISION**	Gross total	9	82%
	Sub total	2	18%
**TUMOR LOCALIZATION**	Posterolateral	5	46%
	Anterolateral	2	18%
	Lateral	2	18%
	Anterior	2	18%

## Results

Surgical approach in foramen magnum meningiomas is important as the mass is close and coherent to neighboring neuro-vascular structures. Surgical and post-operative results are better and total removal rates are higher in the ones with posterior or posterolateral localization. Surgeries of masses with anterior and anterolateral localizations are more difficult, morbidity and mortality rates are higher. Although tumor localization is guiding and yet limiting in terms of determination the form of surgery to be applied, C1 laminectomy in addition to posterior suboccipital craniectomy and far lateral approach including 1/3 condyle resection provide opportunities for gross total tumor excision in nearly all of the cases. Excessive bone tissue loss may be prevented in approaches performed by modifying with suboccipital craniotomy and C1 laminoplasty and cervical instability development may be prevented and post-operative neck pain may be reduced as well as cosmetic positive appearance may be provided in the patients in post-operative period.

## Discussion

The appropriate one from anterior, anterolateral, posterior, posterior far lateral and lateral surgical approach forms is performed on foramen magnum lesions. Surgical interventions to this region are risky in terms of morbidity and mortality [11,12]. Anterior transoral approach may be applied on the anterior localized lesions of this region. Depth and contamination of the operating area, including risks in terms of infections, insufficient lateral exposure and formation of cerebrospinal fluid (CSF) fistula are disadvantages [1,2,13,14]. The areas of operation in anterolateral and posterior approaches are clean. Providing opportunity to decompression and stabilization in C1 and C2 levels at the same time is advantage [15]. Combination of C1 laminectomy with posterior midline suboccipital craniectomy in tumors with posterior or posterolateral localizations is a classic and safe method. Classic suboccipital approach may cause neural and vascular structures to be damaged in patients with lateral extension as well as it is fast and easy [1,16]. Sami *et al*. [17] identified posterior median approach in 40 cases of series, they emphasized that they performed gross total resection in 12 of 15 spinocranial meningioma cases and 13 of 25 craniospinal meningioma cases. Hero [18] identified far lateral approach, George *et al*. [19] identified far lateral modification for vertebral artery aneurysms, and they reported that tumors with anterior localizations were reached more easily. Far lateral approach is difficult and many surgeons are not used to it. Development risks of temporary or permanent lower cranial nerve deficits and CSF fistula are high in far lateral approaches, which are aggressive approaches. When the literature is investigated, it is emphasized that there are high differences such as 2-25% and 6-61% in terms of neurologic morbidity and mortality between posterior median approach and far lateral approach [1,3,20,21-23]. In our own study, we performed, in company with neuro-monitor, C1 laminoplasty with posterior suboccipital craniotomy for 9 of our patients that we received to the operation and 1/3 condyle excision and C1 laminectomy with suboccipital craniectomy by far lateral approach for 2 of our patients. Tumor was apparent in the lateral part of spinal cord in all patients, to whom we applied both surgeries, after the dura was opened. Denticulate ligaments were incised in required cases. Spinal cord and lower cranial were decompressed from the tumor by taking small parts by the help of ultrasonic aspirators. Tumor parts are left in 2 cases, in whom they were coherent to lower cranial nerves and vertebrobasilar complex in an advanced way. Integrity of ventral percentage arachnoid membrane was tried to be protected. Surgery of all our patients continued only in the part of the spinal cord with tumor. At the end, dura, from which tumor was originated, was coagulated as the capsule of the tumor was removed.

In our study, temporary lower cranial nerve (IX, X) involvement and dysphagia developed in 2 of our patients (18%) with anterolateral and anterior localizations, to whom we performed far lateral interventions. Complaints of the patients, whose post-operative oral intakes were limited and steroid was started, regressed after a short period of time. When the literature is investigated, it is reported that there are various temporary and permanent neurological complications (between 6-61%) in far lateral approach [1,16,20,22,23]. Lumbar drainage was started to be applied due to CSF leakage from the wound site on 5th day of post-operative period in 1 case with anterior localization, to whom we performed tumor excision with far lateral approach. CSF leakage was ceased without needing a second surgery by two weeks of follow-up by lumbar drainage. It is reported in various series that CSF fistula is frequently observed in the cases, to whom far lateral approaches are applied [1,8,9,22,23]. Posterior median surgery approach is a safe and effective method for foramen magnum meningiomas, and it is an approach recognized and well-known by the surgeons. There is no need for excision of condyle or lateral mass in this approach. Post-operative recovery is fast. While occipitocervical fusion is rarely needed in posterior approach, fusion between 0% and 66% may be needed in occipital condyle resection in far lateral approach [18,21,24]. Post-operative 50% rate of instability may develop in craniocervical intersection made on one side and condyle excisions more than 1/3 and occipitalcervical fusion may be needed [2]. Surgery made by combining C1 laminectomy to posterior midline suboccipital approach in posterior or posteriolateral localized tumors is an appropriate approach for these region tumors [6,25]. Sohn *et al*. [8] combined midline suboccipital craniotomy and C1 laminoplasty into all cases only with ventral localized foramen magnum in 11 cases of study in 2013. At the same time, they reported that posterior approach will be insufficient and applying far lateral approach will be more correct in the cases, which were thought to have quite high tumor vascularity. We applied this application, which was very rare in literature and performed by Sohn *et al*. [8] to our 7 cases, and we performed gross total tumor excision. VAS values of these patients for post-operative neck pain were quite lower than pre-operative VAS values in the 6th month of follow-ups, and they regressed to 4 in average. On the other hand, neck pains in 2 cases, which were performed far lateral approach, in post-operative 6th month continued in a level to require medical treatment, and no decrease was observed in VAS values. Neck pain is frequent in advanced condyle resection [16,24,26].

## Conclusion

**Figure 5:**
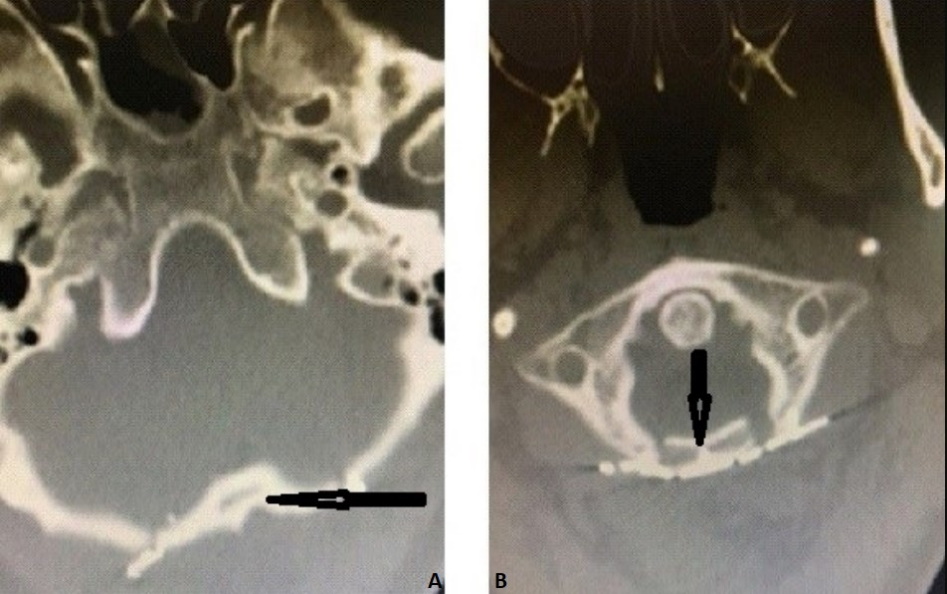
Postoperative cervical axial ct ((A) postoperative the patient's bone removed by suboccipital craniotomy was repositioned with mini-plate and mini-screw and (B) C1 laminoplasty. Black arrow indicates occipital bone and C1 lamina)

The surgical approach in meningiomas of the foramen magnum is important because of adjacency and adherence of the mass to neurovascular formations. Surgical and postoperative results are better in those located posterior or posterolateral sites; and total excision rates are higher. Surgical removal of the masses located on the anterior site is difficult; morbidity and mortality rates are higher. Although tumor location guides and limits the surgical method, posterior suboccipital craniectomy and additional C1 laminectomy approach allow gross total tumor excision. Suboccipital craniotomy bone which was repositioned with mini-plate and mini-screw and C1 laminoplasty may prevent excess bone loss; a positive cosmetic appearance may be achieved and cervical instability may be prevented. (Figure 5). The small sample size of our case study and absence of control group as it is retrospective pose obstacles for generalization of the study we are conducting.

### What is known about this topic

- Surgical removal of the masses located on the anterior site is difficult; morbidity and mortality rates are higher;
- Posterior median surgery approach is a safe and effective method for foramen magnum meningiomas, and it is an approach recognized and well-known by the surgeons.

### What this study adds

- Suboccipital craniotomy bone which was repositioned with mini-plate and mini-screw and C1 laminoplasty may prevent excess bone loss; a positive cosmetic appearance may be achieved and cervical instability may be prevented.

## Competing interests

The authors declare no competing interests.
